# New Hydroxyproline Radiocarbon Dates from Sungir, Russia, Confirm Early Mid Upper Palaeolithic Burials in Eurasia

**DOI:** 10.1371/journal.pone.0076896

**Published:** 2014-01-08

**Authors:** Shweta Nalawade-Chavan, James McCullagh, Robert Hedges

**Affiliations:** Oxford Radiocarbon Accelerator Unit, Research Laboratory for Archaeology and the History of Art, University of Oxford, Oxford, United Kingdom; Illinois State University, United States of America

## Abstract

Sungir (Russia) is a key Mid-Upper Palaeolithic site in Eurasia, containing several spectacular burials that disclose early evidence for complex burial rites in the form of a range of grave goods deposited along with the dead. Dating has been particularly challenging, with multiple radiocarbon dates ranging from 19,160±270 to 28,800±240 BP for burials that are believed to be closely similar in age. There are disparities in the radiocarbon dates of human bones, faunal remains and charcoal found on the floor of burials [1], [2], [3]. Our approach has been to develop compound-specific methods using High Performance Liquid Chromatography (HPLC) to separate single amino acids, such as hydroxyproline, and thereby avoid the known human contamination on the bones themselves. Previously, we applied this technique to obtain radiocarbon dates of ∼30,000 BP for Sungir 2, Sungir 3 and a mammoth bone from the occupation levels of the site [4]. The single amino acid radiocarbon dates were in good agreement with each other compared to all the dates previously reported, supporting their reliability. Here we report new hydroxyproline dates for two more human burials from the same site, Sungir 1 and Sungir 4. All five hydroxyproline dates reported are statistically indistinguishable and support an identical age for the group. The results suggest that compound-specific radiocarbon analysis should be considered seriously as the method of choice when precious archaeological remains are to be dated because they give a demonstrably contaminant-free radiocarbon age. The new ages are, together with the previously dated ‘Red Lady of Paviland’ human in the British Isles, the earliest for Mid Upper Palaeolithic burial behaviour in Eurasia, and point to the precocious appearance of this form of rite in Europe Russia.

## Introduction

**Figure 1:**
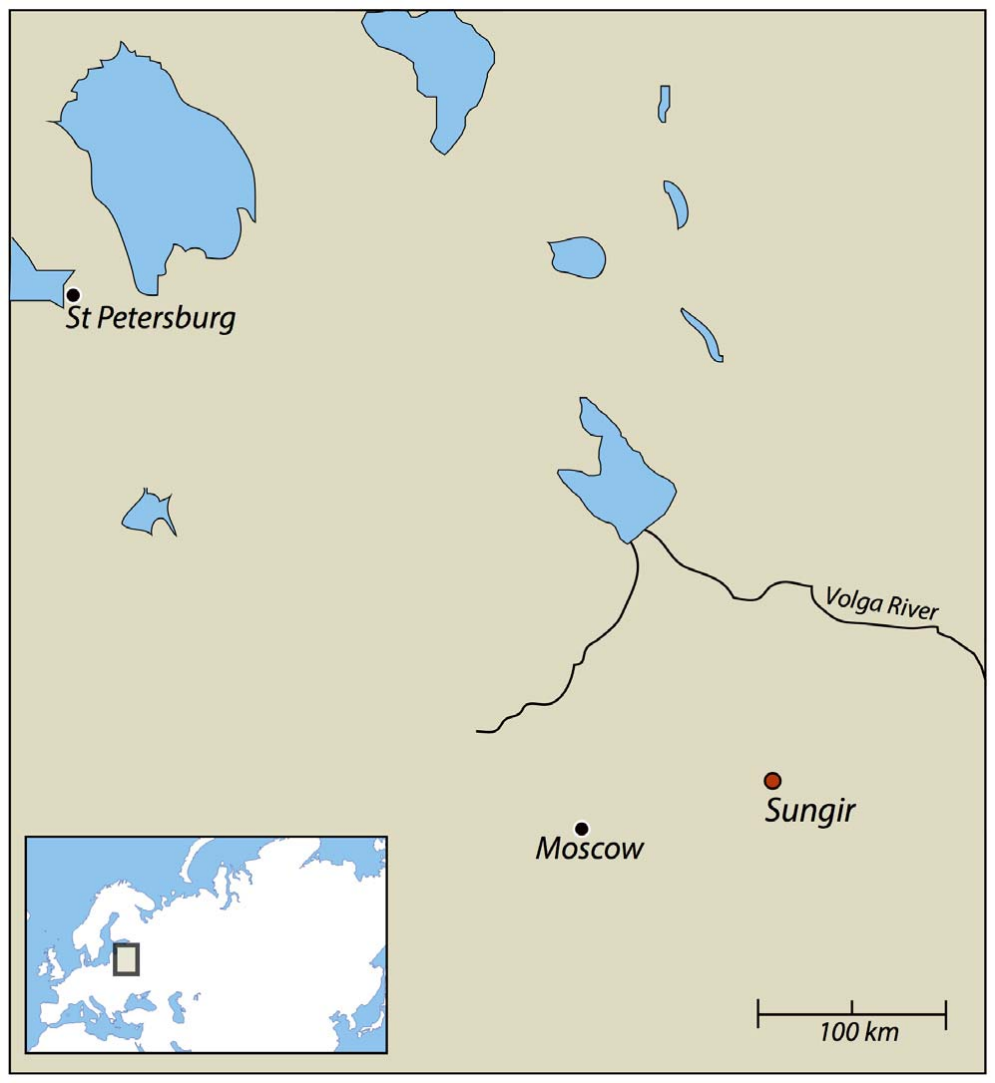
Location map of the Sungir site (56°11′N 40°30′E).

Sungir, Russia is a key early Mid-Upper Palaeolithic site that was discovered in the 1950s. It is situated about 197 km east of Moscow near the modern city of Vladimir (Figure 1). The site is widely known for the presence of up to 8 human individuals, some of whom were interred with a rich material culture, including spears made of mammoth ivory, ivory beads and perforated fox teeth. The remains have been ascribed to the Mid Upper Palaeolithic on the basis of the material culture and lithic evidence, and the presence of ochre, a feature that links the wider corpus of complex human burials during this period, from Russia to Portugal [1]. The lithic evidence has been diagnosed as ‘Streletskian’. This industry includes a range of lithic tools, including triangular bifacial points with concave bases, triangular bifacially thinned points and ‘poplar-leaf’ points of varying width and thickness. Sungir is thought to be a late stage of the Streletskian and characterized by a greater proportion of blades and fewer ‘leaf-points’, with more burins than at other sites [5]. Russian colleagues place the site in a transitional phase related to the previous early Upper Palaeolithic (EUP) [6], [7].

**Figure 2:**
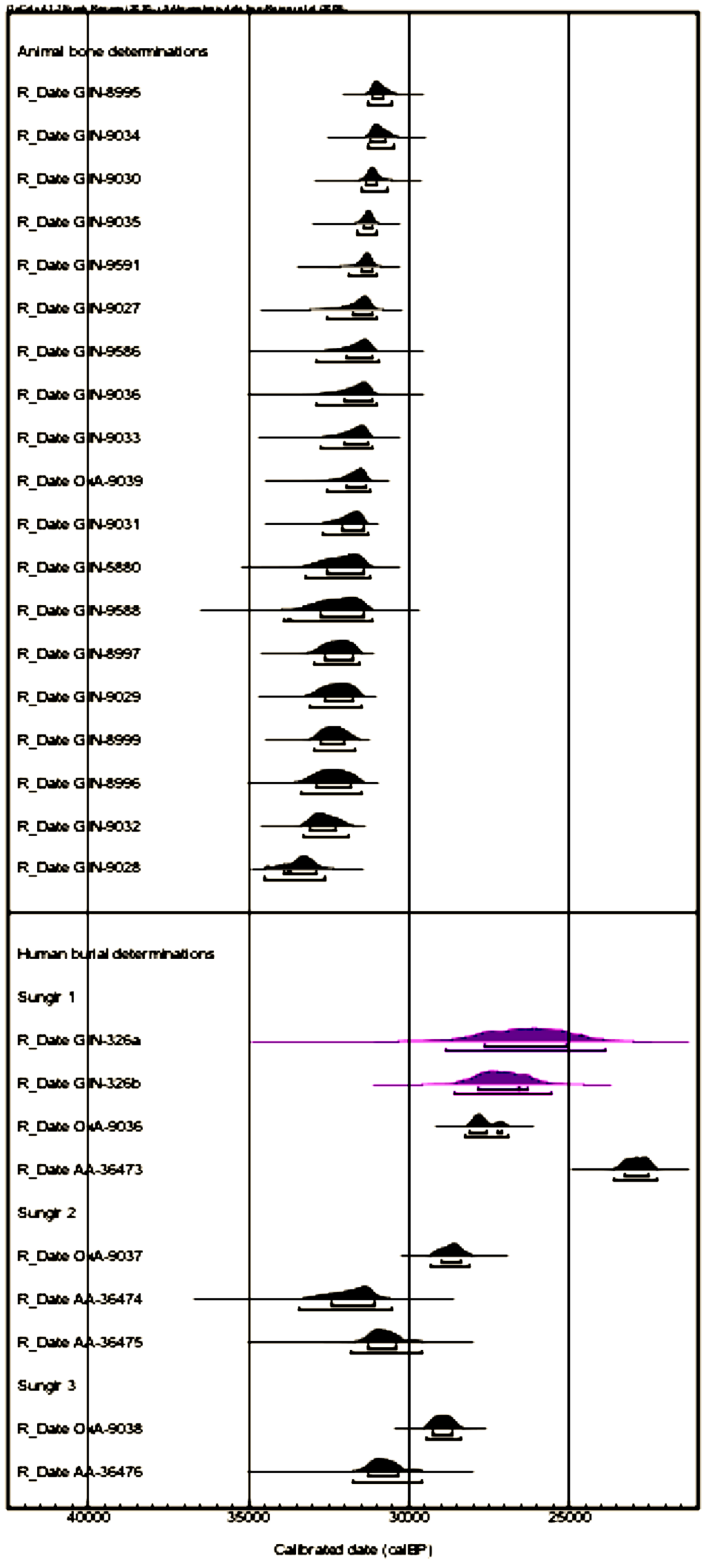
Previous radiocarbon determinations from the site of Sungir (data from Dobrovolskaya et al., 2012). The two dates in purple are charcoal; the remainder is on bone. Note the wide spread in the human bone determinations. Calibration undertaken using INTCAL09 (Reimer et al., 2009).

The radiocarbon dating of carbonaceous material from the site has proven extremely challenging. The current corpus of dates discloses a wide span, at odds with the archaeology of the site (Figure 2). Three laboratories, in Oxford, Arizona and Kiel [1], [2], [3] have obtained direct AMS dates from skeletal material from the site, but the results vary greatly and lack any reproducibility. An analysis of some of the results suggests remaining contamination, indicated by higher than acceptable C∶N atomic ratios where this has been measured. This seems to indicate that the cause of the ages, which range from 19,000 to 27,000 BP, might be the inability of the radiocarbon pretreatment chemistry to isolate a pure collagen fraction. There is much evidence to suggest that the bones have been extensively conserved and curated after excavation, and this is probably the source of the problem [based on two visits to the Gerasimov Laboratory by one of us (TH) and Buzhilova (pers.comm.) and the late T. Belueva (pers.comm.)]. The conservation material consists of a polymer comprising tree sap (termed kanefol) as well as polyvinylbutyral, phenol/formaldehyde and ethanol (pers. obs.) [4].

Recently, Dobrovolskaya et al. [3] obtained dates from Sungir 1 and 3. For Sungir 1, their result was 27,050±210 BP (KIA-27006). They concluded that this was the most reliable age of the corpus so far determined, indicating that previous dates were severely contaminated and not accurate. In addition, the fact that the result broadly overlapped with another date from Sungir 3 (26,000±410 BP KIA-27007) suggested to them that the age of the burials generally was to this period, i.e. ∼26–27 ka BP. The results were argued to be the final word on the age of the site because they appeared in agreement at 2 standard deviations. Afterwards, however, our group obtained new results on Sungir 2 and 3 using single amino acid techniques, specifically dating hydroxyproline (Hyp), and these showed that the determinations of Dobrovolskaya et al. [3] were very likely to be underestimates. In this paper, we present more dates that corroborate the results and conclusions of Marom et al. [4], which further suggests that all previously determined non-Hyp dates are aberrant and much too young.

We selected new material for dating from the Sungir 1 skeleton, a man estimated at ∼50–55 years of age and buried near to the Sungir 2/3 double burial. The skeleton has previously been directly dated at ∼19–23,000 BP in Oxford and Arizona as well as by Dobrovolskaya et al. [3] more recently. As mentioned above, however, Marom et al. [4] had found the age of the double burial to be significantly older, and for this reason we were interested in a direct date of the Sungir 1 skeleton using our HPLC protocol, targeting Hyp once again.

In addition, in the double burial there is a small piece of a human femoral diaphysis (Sungir 4), which was placed into the grave. We also sampled this to add to the corpus of direct dates of human bone from the site, and to compare it to the various radiocarbon ages from the double burial.

## Method and Materials

We obtained material and permission for dating from Dr. E. Balueva (Gerassimov Laboratory, RAS, Moscow, Russia).

### Separation of hydroxyproline using preparative HPLC

The novel compound specific radiocarbon methods developed at the Oxford Radiocarbon Accelerator Unit (ORAU) were used for separating the single amino acids from hydrolysed bone collagen. The method of compound specific dating differs from other macromolecular pre-treatment methods because it separates the compound of interest from the rest of the bone matrix rather than attempt to remove contamination from the bone collagen itself using macromolecular methods [8]. These latter methods may sometimes not be effective depending on the nature of the contamination. Crossed-linked contaminants are difficult to remove using routine methods [9], [10]. As our method involves the hydrolysis of collagen prior to chromatographic separation to break the protein chain into single amino acids, this also effectively breaks the linkage between protein and the contaminants [11], [12], [13].

Samples that are very low in collagen are difficult to be date accurately when collagen is being targeted because higher starting weights of bone are required and this means that often the effect of contamination is multiplied several-fold, leading sometimes to erroneous dates [14]. Hyp dating has an advantage here because larger starting weights may be used without over the bulk collagen dating in low collagen yield samples indicating its significance to be accepted as a more routine method.

Bone samples were sandblasted to clean, collagen was extracted using standard procedures without ultrafiltration [15], [16] and was hydrolysed by treating with excess of 6M hydrochloric acid in a nitrogen atmosphere at 105°C for 24 hr. Hydrochloric acid was removed using a vacuum evaporator and the washed residue was then reconstituted in pH 3 water and was filtered through 0.2μ PTFE syringe filters before injecting onto the chromatographic system equipped with a Primesep A HPLC column.

**Figure 3:**
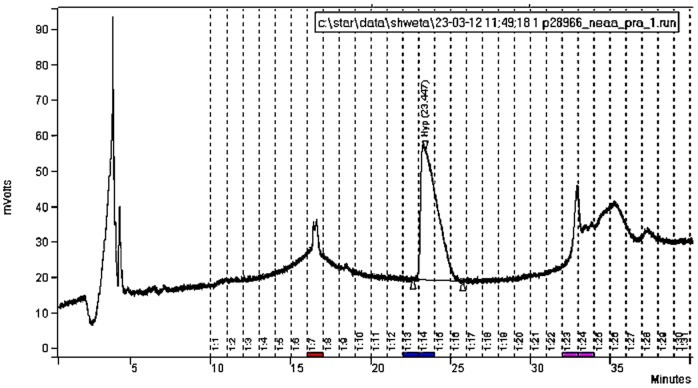
Chromatogram for hydroxyproline separation on mixed mode preparative HPLC using 20 mg collagen hydrolysate of a Sungir sample.

The mixed mode chromatographic method has been improved from Marom et al. [4] by replacing the 0.3% ortho-phosphoric acid gradient mobile phase with Milli-Q™water an isocratic flow rate of 15 ml/min. This modification in the method reduces the chromatographic time significantly as the 2^nd^ injection to remove phosphoric acid from the fractions is not required. The detailed method can be found in Nalawade-Chavan et al. [17]. Although this method can separate all individual essential and non-essential amino acids, for this work, only amino acid Hyp was targeted by injecting the hydrolysed collagen solution directly on to the Primesep A column. The column was washed with 0.3% ortho-phosphoric acid and Milli-Q water to remove remaining, amino acids and to equilibrate column before the next injection. Figure 3 gives sample chromatogram for 20 mg of collagen Hydrolysate for one of the Sungir samples.

The Hyp fractions were evaporated to dryness using a vacuum evaporator, reconstituted in 30 µL of pH 3 water and added to 10 mg of Chromosorb™ in tin capsules prior to combustion. ORAU's routine procedures were followed for combustion, graphitization and AMS dating of the Hyp [18].

The Hyp dates in Table 1 are corrected only for routine procedures such pre-treatment chemistry, combustion, and graphitization necessitating a correction for the extraneous dead and modern carbon added during chromatographic separation using the following correction algorithm.
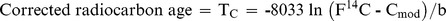

*Where,*

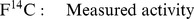


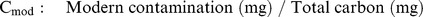


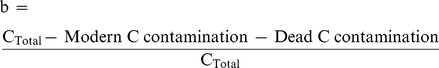


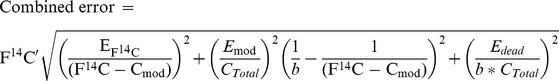

*Where,*

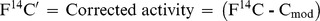


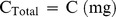


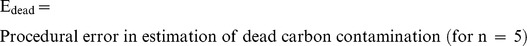


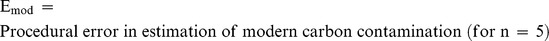


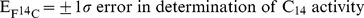

Table 1 illustrates the data for new corrected Hyp radiocarbon dates.

**Table 1:** Data for new Hyp ^14^C dates.

Sample ID	Lab Nr	^14^C age BP (±1σ error)	F^14^C ±	C∶N Ratio	C (mg)	Corrected F^14^C ±	Corrected ^14^C age BP (±1σ error)
Sungir 1	X-2464-12	28650±400	0.02824±0.00145	5.0	0.75	0.02742±0.00143	28890±430
Sungir 4	X-2462-52	29670±289	0.02489±0.00086	5.1	1.37	0.02443±0.00086	29820±280

**Figure 4:**
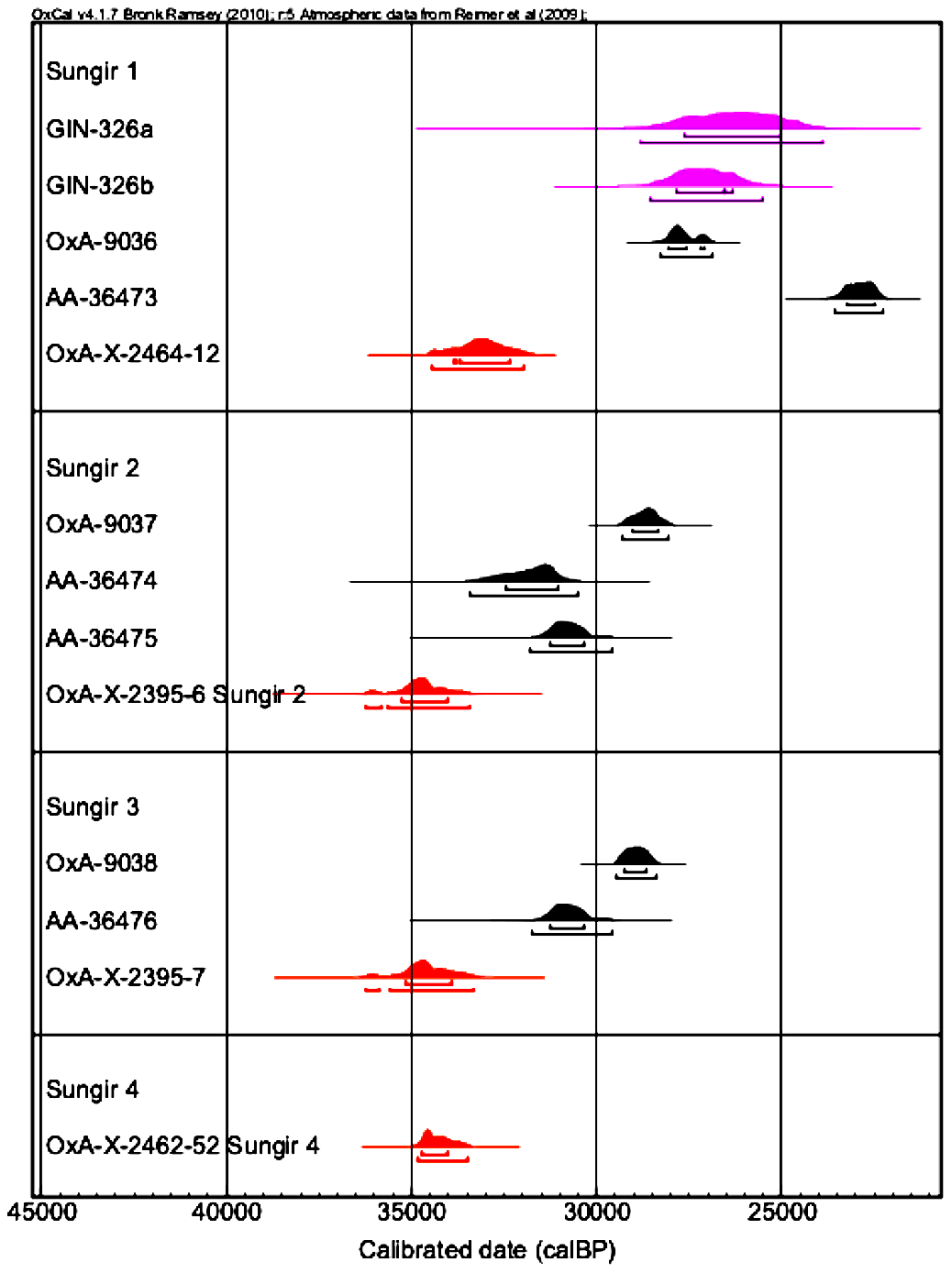
Human bone determinations from Sungir. The calibrated probability distributions in red are those on the Hyp amino acid. Calibration undertaken using INTCAL09 (Reimer et al., 2009).

Figure 4 shows all the calibrated Hyp dates for the Sungir samples that we have obtained, compared with the previous results. The new date for the Sungir 1 burial is once again older than the previously determined results, including the most recently determined date of Dobrovolskaya et al. [3]. We selected what we thought to be bone that is largely free of conservation material, although it is difficult to be absolutely certain. The Hyp result is identical to the dates obtained by Marom et al. [4] on Hyp extracted from the two skeletons in the double-burial, thought to probably be contemporary with Sungir 1. In addition, our new direct date from Sungir 4, the femoral diaphysis excavated in the double burial is identical to the other Hyp dates from the human bones. Taken together, this confirms the results of Marom et al. [4] and gives us an increased level of confidence that these determinations are accurate. The results suggest once more that there is a modern contamination in the bones from Sungir that is derived from museum conservation, and potentially of a high molecular weight and/or cross-linked strongly with the collagen from the bone.

### Fourier Transform Infra-Red (FTIR) spectroscopy

Fourier Transform Infra-Red (FTIR) spectroscopy was used to confirm the presence of consolidants that may have been applied during curation and storage. Infrared spectra of bone powder were obtained using a Varian Excalibur series FTIR with a Specac Golden Gate ATR. Data was processed using Digilab Resolutions Pro 4.0 software. Each sample was run for 64 scans and each spectrum was subjected to background subtraction.

**Figure 5:**
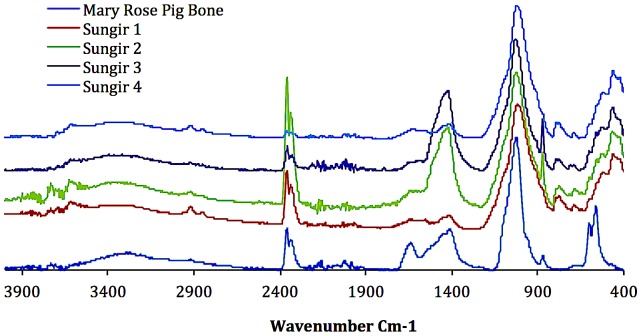
Comparison between FTIR spectra of Sungir human bone samples with FTIR spectra of Mary Rose pig bone, an ORAU in-house standard.

FTIR spectra of the bone samples (Figure 5) were compared with those of a pig rib from the wreck of the Mary Rose (AD 1545), an ORAU in-house standard known to be non-conserved [19].

The FTIR suspect conservation material consists of a polymer comprising tree sap (termed kanefol) as well as polyvinylbutyral, phenol/formaldehyde and ethanol. It appears not to be removed by the pretreatments applied, which included the ultrafiltration protocol. Acid hydrolysis of the collagen and amino acid separation by HPLC is a superior method for this type of highly contaminated material.

### Bayesian modeling

**Figure 6:**
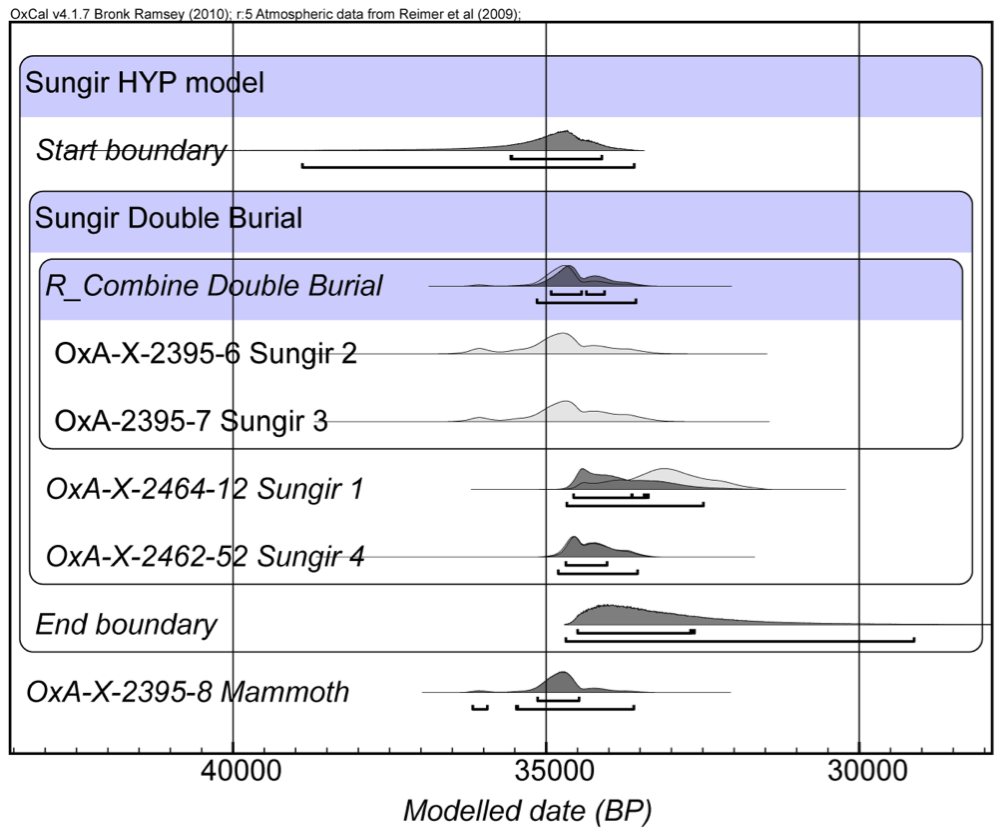
Bayesian age model for the Sungir single amino acid results.

We built a Bayesian age model using OxCal 4.0 [20] to determine a more probabilistic calendar age for the Sungir burials. This is important since it is now well known that interpreting calibrated results by eye is very problematic, so even simple models offer the possibility of obtained more refined and precise calibrated ranges [21]. The model utilizes the archaeological prior knowledge regarding the contemporaneity of the double burial, assuming that the two individuals were interred at the same time, but makes no firm assumptions regarding the relationship between the double burial and the other human remains. We used General t- and S-specific outlier models in the analysis [22]. There were no significant outliers. The results suggest that Sungir was occupied from 38900–33590 cal BP (95% prob.) and prior to 34500–32630 cal BP (Figure 6). The precision on these boundary ranges might be improved with additional dating, there are few dates that are considered reliable enough for this at present. The new Hyp determinations are the oldest radiocarbon dates obtained at the site. We conclude that the previous results, particularly the human bone dates, are too young and widely spread due to conservation products remaining un-removed by previous pretreatment chemistry. The animal bone determinations too are widely ranging, but we are not able to determine with confidence whether they too are aberrantly young. Although the most parsimonious explanation must be that that are, given previous experience, this remains to be tested. The oldest animal bone determinations (Figure 2) previously obtained approach the age we have determined for the Sungir humans, but none is as old at them, or the mammoth date we dated previously using Hyp [4].

## Results and Discussion

**Figure 7:**
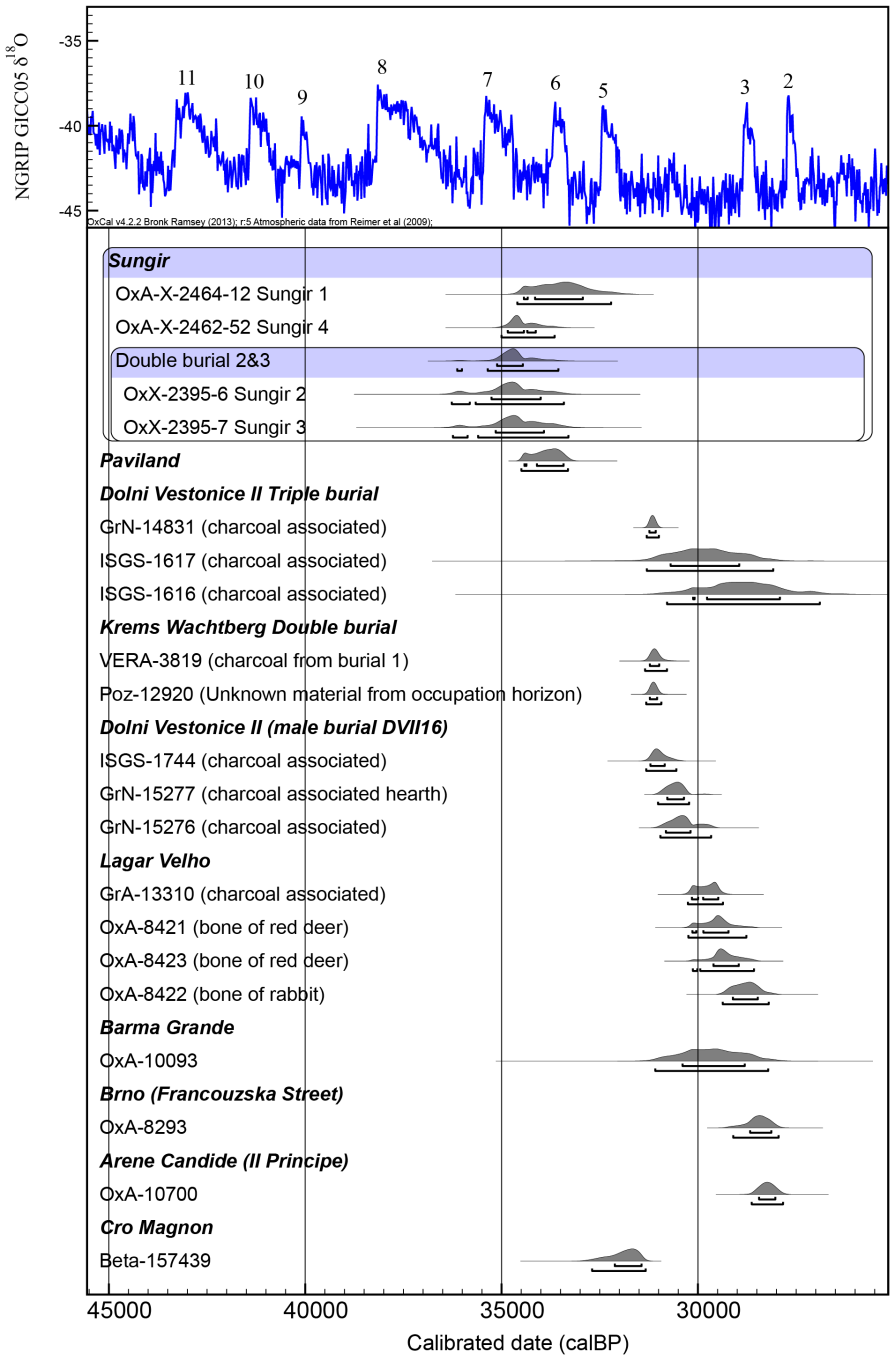
Hyp dates from Sungir compared against other examples of Mid-Upper Palaeolithic burials. These later data are from Jacobi and Higham [23]; Formicola et al. [24]; Henry Gambier [25], Pettitt et al. [26], [27], Pettitt and Trinkaus [28] Svoboda et al. [29] and Einwögerer et al. [30]. Determinations from other sites that are not identified are all on human bone. The Paviland determination is an error weighted mean [23]. Dates are calibrated using the INTCAL09 curve [31] and compared against the NGRIP δ^18^O palaeotemperature record tuned to the Hulu Cave timescale [32]. Numbers on the δ^18^O record represent Greenland Interstadials (GI). See text for details.

We compared the new Sungir ages with other Mid-Upper Palaeolithic burials (Figure 7), including the ‘Red Lady’ of Paviland, Barma Grande, Dolni Vestonice, and Lagar Velho. The comparison shows that the Sungir results, along with the mean of the two ultrafiltered ‘Red Lady’ results, are the very earliest. In previous work, Jacobi and Higham placed the ‘Red Lady’ within the early Mid-Upper Palaeolithic, despite its much older date which places it towards the latter end of sites dating to the Aurignacian technocomplex [23]. The close agreement between the Paviland result and the new Sungir single amino acid dates suggests this was justified. It also suggests the possibility that at least some of the other determinations from elaborate Mid-Upper Palaeolithic burials might be similarly revised under renewed dating attempts using more robust pretreatment methods. These would include the human bone collagen results, but probably not the associated determinations on charcoal (see Figure 7).

## Conclusions

We have produced more reliable, contaminant free single amino acid determinations from human remains excavated at Sungir, Russia. Prior to the new Hyp determinations, the earliest individual thought to date to this period was the ‘Red Lady’ of Paviland in the British Isles dating to ∼29,000 BP [23]. The Sungir results confirm the early appearance of Mid-Upper Palaeolithic complex ritual burial behaviour in Eurasia evidenced previously by the ‘Red Lady’ redating. The results are broadly contemporary within the precision afforded by the dates. Further dating work from other examples is required to explore aspects of the spread of these burial behaviours and confirm the age of some of the previously dated human remains.

The new dates illustrate once again the crucial importance of pretreatment chemistry in the accurate dating of precious samples of rare human bone from this period. For curated and conserved human bones, our contention is that only a single amino acid approach is robust enough to generate reliable results for prehistorians, particularly where modern conservatives have been used to treat and preserve the material.

## References

[1] Pettitt P, Bader N (2000) Direct AMS radiocarbon dates for the Sungir mid Upper Palaeolithic burials. Antiquity 74: 269–270.

[2] Kuzmin Y, Burr G, Jull A, Sulerzhitsky L (2004) AMS 14C age of the Upper Palaeolithic skeletons from Sungir site, Central Russian Plain. Nuclear Instruments and Methods in Physics Research Section B: Beam Interactions with Materials and Atoms 223–224: 731–734.

[3] Dobrovolskaya M, Richards M, Trinkaus E (2012) Direct radiocarbon dates for the Mid Upper Palaeolithic (eastern Gravettian) burials from Sunghir, Russia. Bulletins et Memoires de la Societe d'anthropologie de Paris 24 1–2: 96–102.

[4] Marom A, McCullagh J, Higham T, Sinitsyn A, Hedges R (2012) Single amino acid radiocarbon dating of Upper Palaeolithic modern humans. Proc Natl Acad of Sci U S A 109 18: 6878–6881. 22517758 10.1073/pnas.1116328109 PMC3344984

[5] Bradley B, Anikovich M, Giria E (1996) Early Upper Palaeolithic in the Russian Plain: Streletskayan flaed stone artefacts and technology. Antiquity 69: 989–998.

[6] Sinitsyn A (1996) Kostenki 14 (Markina gora): Data, problems, and perspectives. Préhistoire Européenne 9: 273–313.

[7] Sinitsyn A (2004) Les sépultures de Kostienki: Chronologie, attribution culturelle, rite funéraire. La Spiritualité [Les sépultures de Kostienki: Chronology, cultural attribution, funeral rites: the spirituality]. Actes du Colloque de la Commission 8 de l'UISPP (Paléolithique supérieur) (Liège, 2003), ed Otte M (Études et Recherches Archéologiques de l'Université de Liège 106, Liège, Belgium): 237–244.

[8] van Klinken G, Hedges R (1998) Chemistry strategies for organic ^14^C samples. Radiocarbon 40 1: 51–56.

[9] Collins M, Nielsen-Marsh C, Hiller J, Smith C, Roberts J (2002) The survival of organic matter in bone: a review. Archaeometry 44: 383–394.

[10] Hedges R, van Klinken GJ (1992) A review on current approaches in the pre- treatment of bone for radiocarbon dating by AMS. Radiocarbon 34: 279–291.

[11] Stafford T Jr, Hare P, Currie L, Jull A, Donahue D (1991) Accelerator radiocarbon dating at the molecular level. J Archaeol Sci 18 1: 35–72.

[12] van Klinken G, Bowels A, Hedges R (1994) Radiocarbon dating of peptides isolated from contaminated fossil bone collagen by collagenase digestion and reversed phase chromatography. Geochim Cosmochim Acta 58: 2453–2551.

[13] McCullagh J, Marom A, Hedges R (2010) Radiocarbon dating of individual amino acids from archaeological bone collagen. Radiocarbon 52: 620–634.

[14] Grun R (2006) Direct Dating of Human Fossils. Yearbook of Physical Anthropology 49: 2–48. 10.1002/ajpa.20516 17103430

[15] O'Connell T, Hedges R (1999b) Isotopic comparison of hair and bone: Archaeol analyses. Journal of Archaeol Sci 26 6: 661–665.

[16] O'Connell T, Hedges R, Healey M, Simpson A (2001) Isotopic comparison of hair, nail and bone: Modern analyses. Journal of Archaeol Sci 28 11: 1247–1255.

[17] Nalawade-Chavan S, McCullagh J, Hedges R, Bonsall C, Boroneant A, et al (2013) Compound Specific Radiocarbon Dating of essential and non-essential amino acids: Towards determination of dietary reservoir effects in humans. Radiocarbon 55 2–3: 709–719.

[18] Brock F, Higham T, Ditchfield P, Ramsey C (2010) Current pre-treatment methods for AMS radiocarbon dating at the Oxford Radiocarbon Accelerator Unit (ORAU). Radiocarbon 52 1: 103–12.

[19] Bronk Ramsey C, Higham T, Bowles A, Hedges R (2004) Improvements to the pre- treatment of bone at Oxford. Radiocarbon 46 1: 155–163.

[20] Bronk Ramsey C (2009a) Bayesian analysis of radiocarbon dates. Radiocarbon 51 1: 337–360.

[21] Higham T, Basell L, Jacobi R, Wood R, Bronk Ramsey C, et al (2012) Testing models for the beginnings of the Aurignacian and the advent of figurative art and music: The radiocarbon chronology of Geißenklösterle,. Journal of Human Evolution 62 6: 664–676. 22575323 10.1016/j.jhevol.2012.03.003

[22] Bronk Ramsey C (2009b) Dealing with outliers and offsets in radiocarbon dating. Radiocarbon 51 3: 1023–1045.

[23] Jacobi R, Higham T (2008) The “Red Lady” ages gracefully: new ultrafiltration AMS determinations from Paviland. Journal of Human Evolution 55: 898–907. 18929395 10.1016/j.jhevol.2008.08.007

[24] Formicola V, Pettitt P, Del Lucchese A (2004) A direct AMS radiocarbon date on the Barma Grande 6 Upper Palaeolithic skeleton. Curr Anthropol 45 1: 114–118.

[25] Henry-Gambier D (2002) Les fossiles de Cro-Magnon (Les Eyzies-de-Tayac, Dordogne): nouvelles données sur leur position chronologique et leur attribution culturelle. Paleo 14: 201–204.

[26] Pettitt P, van der Plicht J, Bronk Ramsey C, Monge-Soares A, Zilhão J (2002) The radiocarbon chronology. In: Zilhão, J., Trinkaus, E. (Eds.), Portrait of the Artist as a Child: The Gravettian Human Skeleton from the Abrigo do Lagar Velho and its Archaeological Context. Ministéria da Cultura/Instituto Português de Arqueologia, Lisbon: 132–138.

[27] Pettitt P, Richards M, Maggi R, Formicola V (2003) The Gravettian burial known as the Prince (“Il Principe”): new evidence for his age and diet. Antiquity 77 295: 15–19.

[28] Pettitt P, Trinkaus E (2000) Direct radiocarbon dating of the Brno 2 Gravettian human remains. Anthropologie (Brno) 38 2: 149–150.

[29] Svoboda J, van der Plicht J, Kuželka V (2002) Upper Palaeolithic and Mesolithic human fossils from Moravia and Bohemia (Czech Republic): some new ^14^C dates. Antiquity 76 294: 957–962.

[30] Einwögerer T, Händel M, Neugebauer-Maresch C, Simon U, Steier P, et al (2009) 14C dating of the Upper Paleolithic site at Krems-Wachtberg, Austria. Radiocarbon 51 2: 847–855.

[31] Reimer P, Baillie M, Bard E, Bayliss A, Beck J, et al (2009) IntCal09 and Marine09 radiocarbon age calibration curves, 0–50,000 years cal BP. Radiocarbon 51 4: 1111–1150.

[32] Andersen K, Svensson A, Johnsen S, Rasmussen S, Bigler M, et al (2006) The Greenland ice core chronology 2005, 15–42 ka. Part 1: constructing the time scale. Quat Sci Reviews 25 23–24: 3246–3257.

